# YHMI: a web tool to identify histone modifications and histone/chromatin regulators from a gene list in yeast

**DOI:** 10.1093/database/bay116

**Published:** 2018-10-29

**Authors:** Wei-Sheng Wu, Hao-Ping Tu, Yu-Han Chu, Torbjörn E M Nordling, Yan-Yuan Tseng, Hung-Jiun Liaw

**Affiliations:** 1 Department of Electrical Engineering, National Cheng Kung University, Tainan, Taiwan; 2 Department of Mechanical Engineering, National Cheng Kung University, Tainan, Taiwan; 3 Center for Molecular Medicine and Genetics, Wayne State University, School of Medicine, Detroit, MI, USA; 4 Department of Life Sciences, National Cheng Kung University, Tainan, Taiwan

## Abstract

Post-translational modifications of histones (e.g. acetylation, methylation, phosphorylation and ubiquitination) play crucial roles in regulating gene expression by altering chromatin structures and creating docking sites for histone/chromatin regulators. However, the combination patterns of histone modifications, regulatory proteins and their corresponding target genes remain incompletely understood. Therefore, it is advantageous to have a tool for the enrichment/depletion analysis of histone modifications and histone/chromatin regulators from a gene list. Many ChIP-chip/ChIP-seq datasets of histone modifications and histone/chromatin regulators in yeast can be found in the literature. Knowing the needs and having the data motivate us to develop a web tool, called Yeast Histone Modifications Identifier (YHMI), which can identify the enriched/depleted histone modifications and the enriched histone/chromatin regulators from a list of yeast genes. Both tables and figures are provided to visualize the identification results. Finally, the high-quality and biological insight of the identification results are demonstrated by two case studies. We believe that YHMI is a valuable tool for yeast biologists to do epigenetics research.

## Introduction

Histone modification and chromatin remodelling play an important role in DNA replication, transcription and DNA repair (1–3). The N-terminal and C-terminal tails of histones are subject to post-translational modifications, including acetylation, methylation, phosphorylation and ubiquitination (4, 5). Several lines of evidence have shown that these modified histones provide the binding sites for effector proteins to elicit specific and selective effects on these biological processes (1, 6). To date, a large number of domains within these effector proteins that can be associated with acetylated, methylated and phosphorylated histones have been characterized. For example, the bromodomain binds to acetylated histones (7); the BRCA1 C Terminus (BRCT) domain binds to the phosphorylated histones (8); and the plant homeodomain (PHD), chromodomain and tudor domains associate with methylated histones (9–14). A recent study further revealed that tandem PHD fingers of MORF/MOZ acetyltransferases display selectivity for acetylated histone H3 (15). Many chromatin-associated proteins themselves contain these domains or are partnered with effector proteins containing these domains. Therefore, multivalent and combinatorial interactions are likely to be an important aspect of how these chromatin-associated proteins work—a concept known as the histone code hypothesis (1, 6). However, how these multivalent and combinatorial interactions contribute to various biological processes in response to environmental stimuli remains incompletely understood.

In yeast *Saccharomyces cerevisiae*, histones are modified by various histone modification complexes, including Spt-Ada-Gcn5-Acetyltransferase (SAGA), NuA4, COMPASS, Set2 and Dot1 (3, 4). SAGA and NuA4 complexes are able to acetylate histone H3 and histone H4 (H4ac), respectively (3, 16, 17). The COMPASS complex, Set2 and Dot1 are able to methylate lysine 4 of histone H3 (H3K4me), lysine 36 of histone H3 (H3K36me) and lysine 79 of histone H3 (H3K79me), respectively (3). Information about the other histone modifications and their related enzymes is listed in Table 1. The chromatin remodelling complexes include SWI/SNF, RSC, ISWI, CHD, INO80 and SWR1 that are able to slide, displace and exchange histones (3). The histone modification and chromatin remodelling complexes often form a large complex with many subunits containing bromodomain, chromodomain or PHD domain. Therefore, it is likely that the combination of these domains plays an important role in selectivity and specificity of these complexes to their target genes. There are about 400 proteins that regulate gene transcription (18). Therefore, many open questions remain. How many regulators and specific histone modifications are needed to transcribe a subset of genes in response to environmental stimuli? Are there combination patterns of these domains and their corresponding modified histones in order to transcribe a subset of genes?

**Table 1:** Many known histone modifications in *S. cerevisiae* are provided. Most of the information in this table came from Rando and Winston (3)

**Histone**	**Residue**	**Modification**	**Modification enzyme**
H2A	K5	Ac	Esa1, Rpd3
	S129	Ph	Mec1, Tel1, Pph3
H2B	K123	Ub	Rad6, Ubp8
H3	R2	Me	
	K4	Me, Ac	Set1, Jhd2, Rtt109, Gcn5
	K9	Ac	Gcn5, Rpd3, Hos2, Hda1
	S10	Ph	Snf1
	K14	Ac	Gcn5, Rpd3, Hos2, Hda1
	K18	Ac	Gcn5, Rpd3, Hos2, Hda1
	K23	Ac	Gcn5, Rpd3, Hos2, Hda1
	K27	Ac	
	K36	Me	Set2, Rph1, Jhd1
	K56	Ac	Rtt109, Hst3, Hst4
	K79	Me	Dot1
H4	R3	Me	
	K5	Ac	Esa1, Rpd3, Hos2
	K8	Ac	Esa1, Rpd3, Hos2
	K12	Ac	Esa1, Rpd3, Hos2
	K16	Ac	Esa1, Sas2, Sir2, Hos2, Hst1
	K20	Me	

**Figure 1:**
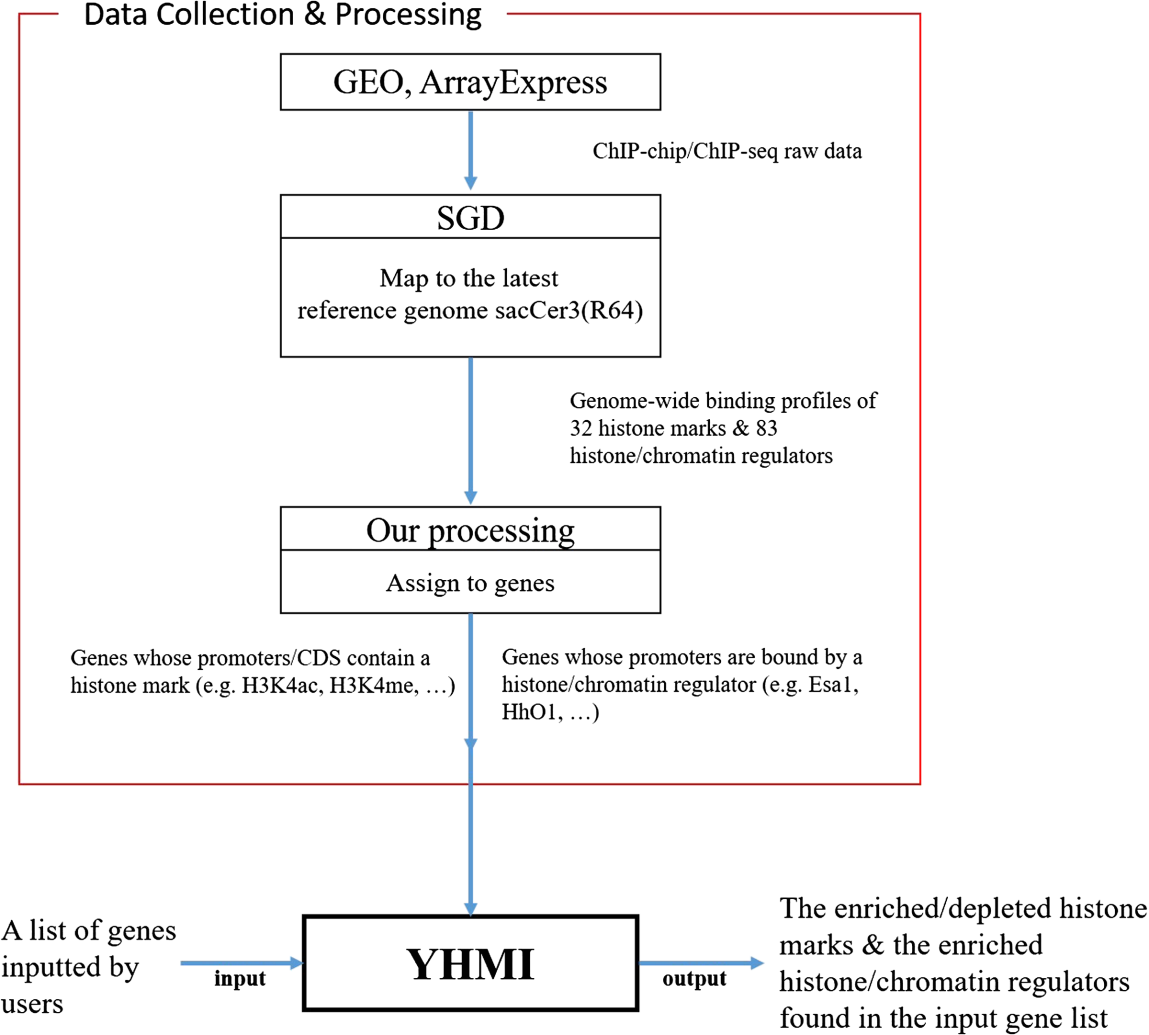
YHMI includes the ChIP-chip/ChIP-seq datasets of 32 histone marks and 83 histone/chromatin regulators.

**Table 2:** The information about the collected ChIP-chip/ChIP-seq datasets of histone modifications (15 histone acetylation, 13 histone methylation, 2 histone phosphorylation, 1 histone ubiquitination and 1 histone variant). See Supplementary Table 1 for details

**Type of histone modification**	**Data value**	**Type of histone modification**	**Data value**
Acetylation (H2AK5ac)^a^	log_2_(H2AK5ac/Input^b^)	Methylation (H3R2me2a)^c^	log_2_(H3R2me2a/H3)
Acetylation (H3K4ac)	log_2_(H3K4ac/H3)	Methylation (H3K4me)	log_2_(H3K4me/H3)
Acetylation (H3K9ac)	log_2_(H3K9ac/H3)	Methylation (H3K4me2)	log_2_(H3K4me2/H3)
Acetylation (H3K14ac)	log_2_(H3K14ac/H3)	Methylation (H3K4me3)	log_2_(H3K4me3/H3)
Acetylation (H3K14ac [H_2_O_2_]^d^)	log_2_(H3K14ac/H3)	Methylation (H3K36me)^a^	log_2_(H3K36me/Input)
Acetylation (H3K18ac)^a^	log_2_(H3K18ac/Input)	Methylation (H3K36me2)^a^	log_2_(H3K36me2/Input)
Acetylation (H3K23ac)^a^	log_2_(H3K23ac/Input)	Methylation (H3K36me3)	log_2_(H3K36me3/H3)
Acetylation (H3K27ac)^a^	log_2_(H3K27ac/Input)	Methylation (H3K79me)^a^	log_2_(H3K79me/Input)
Acetylation (H3K56ac)^a^	log_2_(H3K56ac/Input)	Methylation (H3K79me2)	MAT^e^ score (H3K79me2/Input)
Acetylation (H4ac)	log_2_(H4ac/H3)	Methylation (H3K79me3)	MAT score (H3K79me3/Input)
Acetylation (H4ac [H_2_O_2_]^d^)	log_2_(H4ac/H3)	Methylation (H4R3me)^a^	log_2_(H4R3me/Input)
Acetylation (H4K5ac)^a^	log_2_(H4K5ac/Input)	Methylation (H4R3me2s)^a^	log_2_(H4R3me2s/Input)
Acetylation (H4K8ac)^a^	log_2_(H4K8ac/Input)	Methylation (H4K20me)^a^	log_2_(H4K20me/Input)
Acetylation (H4K12ac)^a^	log_2_(H4K12ac/Input)	Phosphorylation (H2AS129ph)^a^	log_2_(H2AS129ph/Input)
Acetylation (H4K16ac)^a^	log_2_(H4K16ac/Input)	Phosphorylation (H3S10ph)^a^	log_2_(H3S10ph/Input)
Histone Variant (H2AZ)	log_2_(H2AZ/H2B)	Ubiquitination (H2BK123ub)	MAT score (H2BK123ub/Input)

a ^a^This is a ChIP-seq dataset.

b ^b^`Input’ means the control experiment, which is the ChIP-chip/ChIP-seq experiment without using any anti-histone modification (e.g. anti-H3K79me2) antibody.

c ^c^We used the ChIP-chip dataset mapped to the plus strand.

d ^d^The yeast cells are grown in the rich medium adding H_2_O_2_.

e ^e^MAT stands for Model-based Analysis of Tiling-arrays (48), which is an algorithm for reliably detecting enriched regions. The higher the MAT score, the higher the enrichment.

Previous studies have produced several valuable genome-wide ChIP-chip/ChIP-seq datasets of histone modifications and binding occupancy of histone/chromatin regulators in yeast *Saccharomyces cerevisiae* to facilitate epigenetics research (18–24). Since these datasets are scattered across the literature, several resources have been developed to provide histone modification information in yeast*.* For example, the Saccharomyces Genome Database (SGD) comprehensively collects the yeast histone modification datasets from the literature and allows users to visualize various histone modifications using JBrowse (a genome browser) (25). The Yeast Nucleosome Atlas (YNA) implements a tool for users to retrieve a list of yeast genes whose promoters and/or coding regions contain a specific combination of histone modifications (26). The ChromatinDB implements a tool for users to analyze specific histone modifications from the input gene list (27). These three resources altogether greatly help yeast biologists to do epigenetics research. Unfortunately, ChromatinDB is no longer available since 2014. Yeast biologists now are lacking a convenient tool to identify the enriched/depleted histone modifications in their gene lists routinely generated from high-throughput experimental technologies (e.g. microarray or next-generation sequencing).

To fill this gap, we developed a web tool called Yeast Histone Modification Identifier (YHMI). YHMI uses ChIP-chip/ChIP-seq datasets of 32 histone modifications (15 histone acetylation, 13 histone methylation, 2 histone phosphorylation, 1 histone ubiquitination and 1 histone variant) and 83 histone/chromatin regulators (18–24). When a user inputs a gene list, YHMI will identify the enriched/depleted histone modifications in the promoters/coding regions and the enriched histone/chromatin regulators in the promoters of the genes in the input list. The identification results are shown both in tables and figures. Therefore, YHMI can be used to shed light on what is unknown in a gene list of interest. Several possible biological questions could be answered by YHMI. For example, what are the enriched/depleted histone codes in a gene list of a specific property (e.g. highly transcribed genes, stress-responsive genes or genes in a specific pathway)? What are the enriched/depleted histone codes in a gene list associated with a specific factor (e.g. target genes of a transcription factor, lipid-binding proteins or hexose transporter genes)?

**Figure 2:**
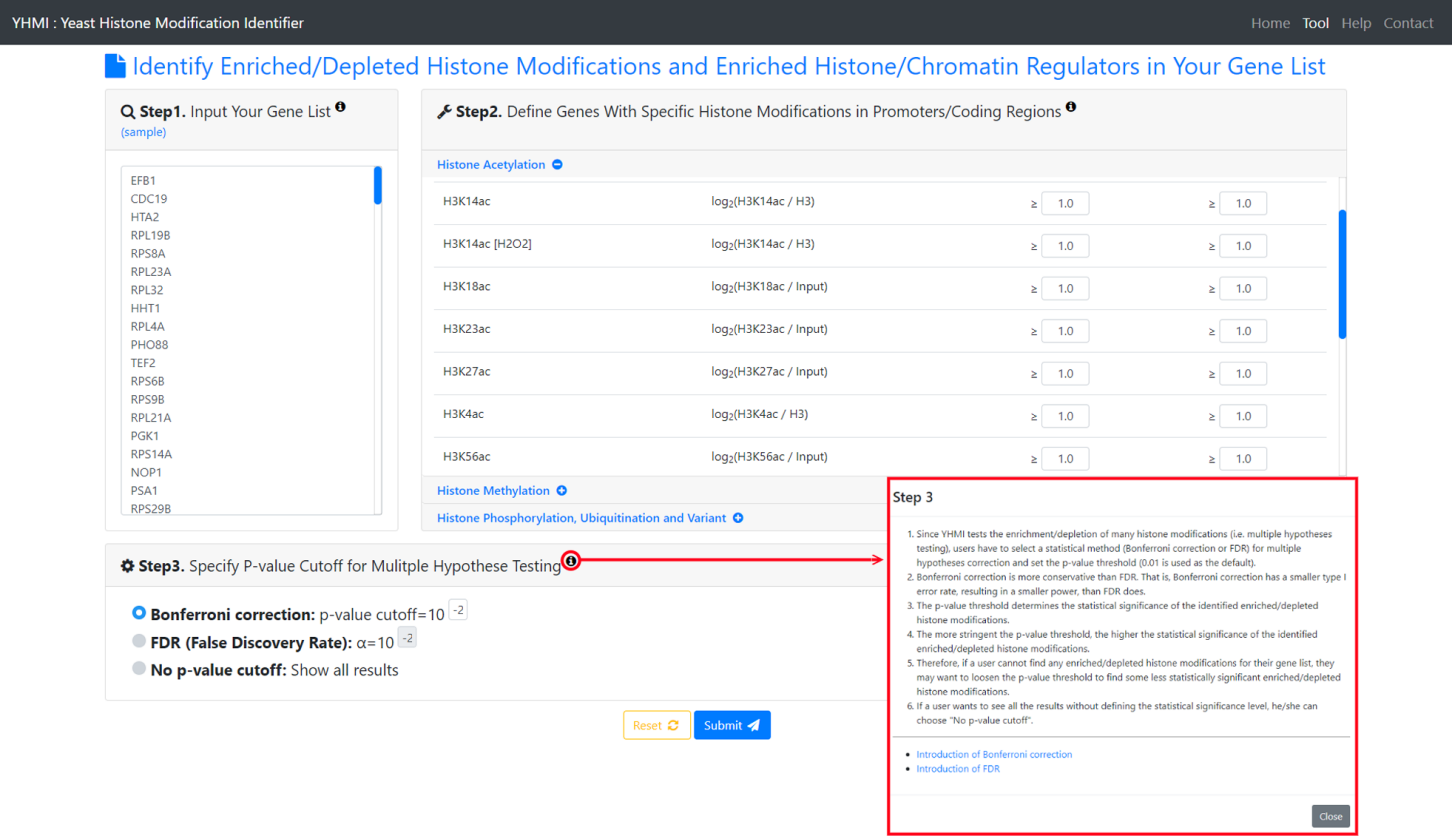
The input page. To use YHMI, users have to go through a three-step process.

**Figure 3:**
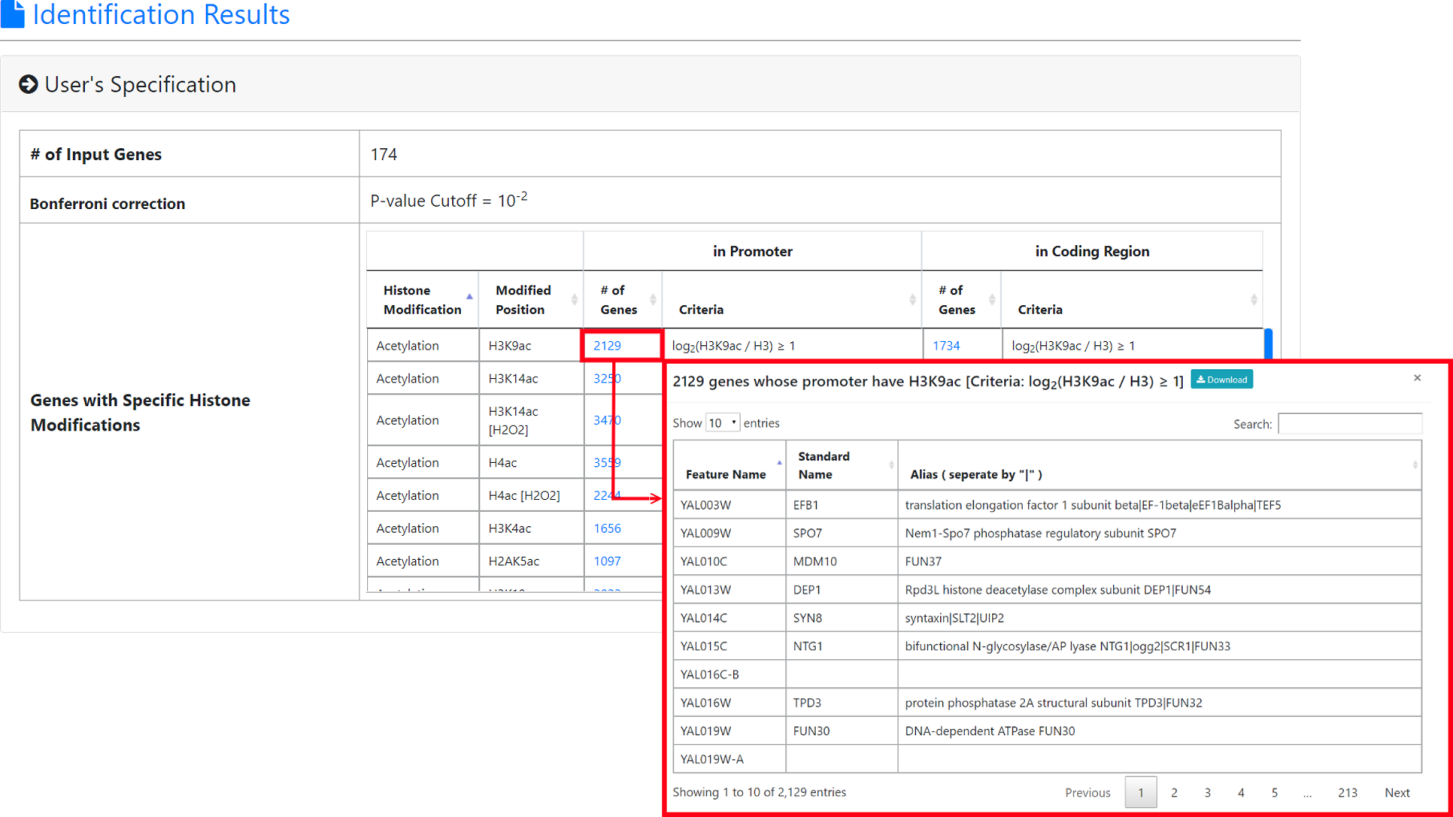
The result page (first part). The first part of the result page contains the information of the user’s settings. Uniquely, users can download all the sets of genes containing specific histone modifications defined by the users for further investigation.

## Construction and contents

### Collection of ChIP-chip/ChIP-seq datasets of histone modifications, histone regulators and chromatin regulators

All the ChIP-chip/ChIP-seq data (18–24) used in YHMI were downloaded from SGD (Figure 1). SGD (25) collected the raw data of ChIP-chip/ChIP-seq from GEO (28) and ArrayExpress (29), mapped the raw data to the latest yeast reference genome sacCer3 (R64) and allowed everyone to download the processed data. Therefore, we directly downloaded the ChIP-chip/ChIP-seq datasets of 32 histone modifications (Table 2 and Supplementary Table 1 for more details about the strain, reference genome, original data source, etc.) and 83 histone/chromatin regulators (Supplementary Table 2) from SGD.

**Figure 4:**
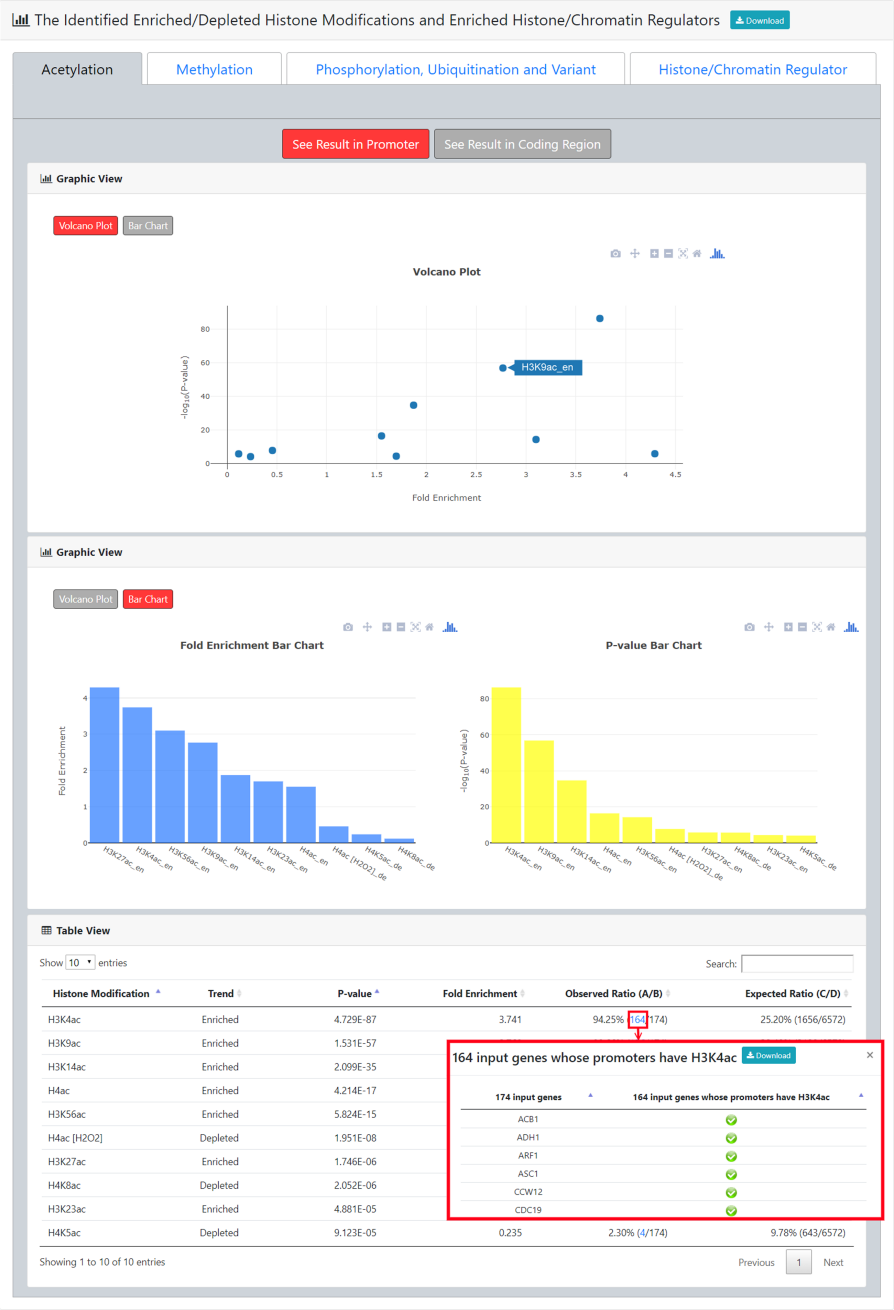
The result page (second part). The second part of the result page provides tables and figures to show the identified enriched/depleted histone modifications (acetylation, methylation, phosphorylation, ubiquitination and histone variant) in the promoters/coding regions of the input gene list. The table contains the name of the histone modification, trend (enriched/depleted), *P*-value, fold enrichment, observed ratio and expected ratio. Moreover, two kinds of figures (a volcano plot and two-bar charts) are also provided for visualization.

### Defining genes whose promoters/coding regions contain a specific histone modification

Following previous studies (19, 23), a gene’s promoter is defined as the region between 500 bp upstream and 100 bp downstream of the start codon. A gene’s coding region is defined as the region between the start codon and the stop codon. The procedure of defining a set of genes whose promoters/coding regions contain a specific histone modification (e.g. H3K4ac) is as follows. First, for each of the 6572 genes in the yeast genome, we extracted the maximal data value (${\mathit{log}}_2\left(\mathrm{H}3\mathrm{K}4\mathrm{ac}/\mathrm{H}3\right)$ in this case) in its promoter/coding region from the corresponding ChIP-chip/ChIP-seq dataset (Table 2). Second, the promoter/coding region of a gene is said to contain H3K4ac if it satisfies ${\mathit{log}}_2\left(\mathrm{H}3\mathrm{K}4\mathrm{ac}/\mathrm{H}3\right)\ge threshold,$ where the threshold is set by the user. For example, when the threshold is set to one, 1656 genes’ promoters and 977 genes’ coding regions are said to contain H3K4ac.

**Figure 5:**
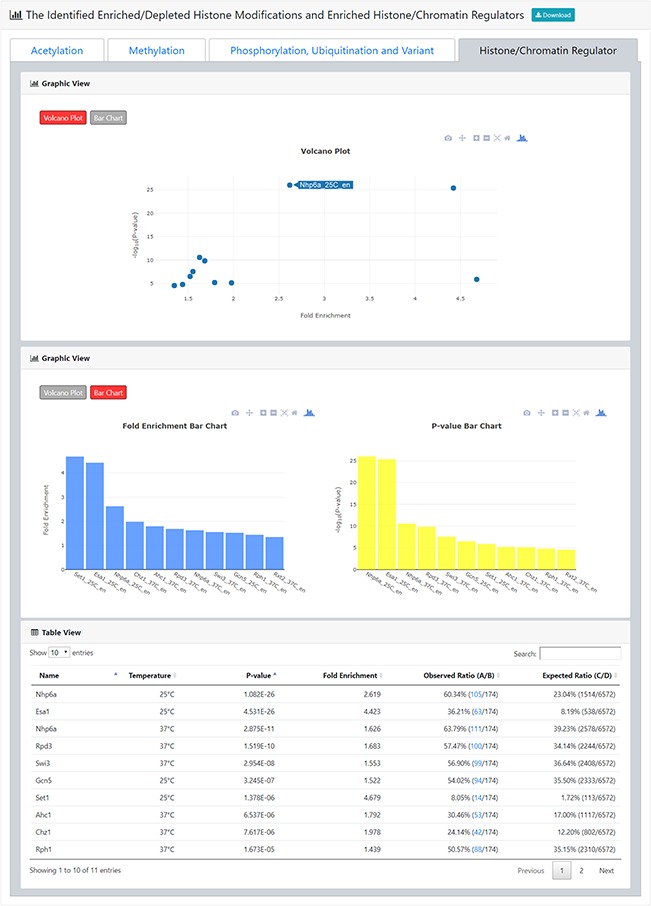
The result page (third part). The third part of the result page provides tables and figures to show the identified enriched histone/chromatin regulators in the promoters of the input gene list. The table contains the name of the histone/chromatin regulator, temperature, *P*-value, fold enrichment, observed ratio and expected ratio. Moreover, two kinds of figures (a volcano plot and two-bar charts) are also provided for visualization.

### Defining genes whose promoters are bound by a specific histone/chromatin regulator

Venters *et al.* (18) identified high-confident [less than 5% false discovery rate (FDR)] interactions between a specific histone/chromatin regulator and genomic DNA (in the yeast genome) under normal (25°C) and acute heat-shock conditions (37°C) by ChIP-chip experiments. Using Venters *et al.*’s results (18) and based on the definition of a gene’s promoter region (see the previous subsection), we can determine the genes whose promoters are bound by a specific histone/chromatin regulator. Supplementary Table 2 provides the names of these 83 histone/chromatin regulators and their target genes.

### Identifying the enriched/depleted histone modifications and the enriched histone/chromatin regulators for the user’s input genes

The main functionality of YHMI is to identify the enriched/depleted histone modifications and the enriched histone/chromatin regulators for the user’s input genes. The procedure for checking whether a specific histone modification (e.g. H3K4ac) is enriched/depleted in the promoters of the user’s input genes is as follows. Let *S* be the set of genes whose promoters contain the histone modification H3K4ac (see the subsection before the previous one for details), *R* be the set of the user’s input genes, $T=S\cap R$ be the set of genes whose promoters contain H3K4ac and are also in the set of the user’s input genes and *F* be the set of all genes in the yeast genome. Then H3K4ac is said to be enriched/depleted in the promoters of the user’s input genes if the observed ratio (|*T*|/|*R*|) in the input genes is significantly higher/lower than the expected ratio (|*S*|/|*F*|) in the yeast genome. |*S*| stands for the number of genes in the set *S* and |*F*| = 6572. The statistical significance is calculated using the hypergeometric testing (30) as follows. The ${P}_{value}(enrichment)$ and ${P}_{value}(depletion)$ for rejecting the null hypothesis (H_0_: H3K4ac is not enriched/depleted in the promoters of the user’s input genes) are calculated as

\begin{array}{c}{P}_{value}(enrichment)=P\left(x\ge \left|T\right|\right)=\sum\limits_{x=\left|T\right|}^{\min \left(\left|S\right|,\left|R\right|\right)}\frac{\left(\begin{array}{@{}c@{}}\left|S\right|\\ {}x\end{array}\right)\left(\begin{array}{@{}c@{}}\left|F\right|-\left|S\right|\\ {}\left|R\right|-x\end{array}\right)}{\left(\begin{array}{@{}c@{}}\left|F\right|\\ {}\left|R\right|\end{array}\right)},\\ {}{P}_{value}(depletion)=P\left(x\le \left|T\right|\right)=\sum\limits_{x=0}^{\left|T\right|}\frac{\left(\begin{array}{@{}c@{}}\left|S\right|\\ {}x\end{array}\right)\left(\begin{array}{@{}c@{}}\left|F\right|-\left|S\right|\\ {}\left|R\right|-x\end{array}\right)}{\left(\begin{array}{@{}c@{}}\left|F\right|\\ {}\left|R\right|\end{array}\right)}\end{array}

where $\left|S\right|$ means the number of genes in set *S* and $$\left(\begin{array}{@{}c@{}}\left|F\right|\\ {}\left|R\right|\end{array}\right)$$ is a binomial coefficient. The ${P}_{value}(enrichment)$ and ${P}_{value}(depletion)$ are then corrected by the Bonferroni correction or the FDR to represent the true alpha level in the multiple hypotheses testing. Finally, H3K4ac is said to be enriched/depleted in the promoters of the user’s input genes if the corrected ${P}_{value}(enrichment)$ or corrected ${P}_{value}(depletion)$ is less than the user-defined threshold. Note that Bonferroni correction and FDR are two statistical methods for multiple hypotheses correction. Bonferroni correction is more conservative than FDR. That is, Bonferroni correction has a smaller type I error rate, resulting in a smaller power, than FDR does.

The procedure for checking whether a specific histone modification is enriched/depleted in the coding regions of the user’s input genes is the same as mentioned above except for the definitions of two terms. Now *S* becomes the set of genes whose coding regions contain the histone modification H3K4ac and $T=S\cap R$ becomes the set of genes whose coding regions contain H3K4ac and are also in the set of the user’s input genes.

The procedure for checking whether a specific histone/chromatin regulator (e.g. Esa1) is enriched in the promoters of the user’s input genes is the same as mentioned above except for the definitions of two terms. Now *S* becomes the set of genes whose promoters are bound by Esa1 and $T=S\cap R$ becomes the set of genes whose promoters are bound by Esa1 and are also in the set of the user’s input genes.

### Implementation and maintenance of the web interface of YHMI

Figure 1 illustrates the overall configuration of YHMI. The web interface of YHMI was developed in Python using the Django MTV framework. The processed histone modification data were deposited in MySQL. All tables, volcano plots and bar charts were produced by the JavaSscript and feature-rich JavaScript libraries (jQuery, DataTables and Plotly.js) to visualize data on the webpage. We also provide the command line version of YHMI (written in Python) for users who want to run YHMI on their local computers (see the Help page of YHMI website). YHMI will be maintained by our lab’s research assistants and we have a backup site (http://cosbi5.ee.ncku.edu.tw/YHMI/). Therefore, the long-term stability of YHMI is guaranteed. In the future, we will keep updating YHMI once new histone modification datasets become available in the literature.

**Table 3:** YHMI successfully identifies most known histone modifications of highly transcribed genes

**Type of histone modification**	**Identified histone modification**	**Identified enzyme known to achieve this histone modification**	**Literature evidence**
Acetylation	H3K4ac enriched in promoter	Gcn5	Guillemette *et al.* (19)
Acetylation	H3K4ac enriched in coding region	Gcn5	Guillemette *et al.* (19)
Acetylation	H3K9ac enriched in promoter	Gcn5	Pokholok *et al.* (20)
Acetylation	H3K14ac enriched in promoter	Gcn5	Pokholok *et al.* (20)
Acetylation	H4ac enriched in promoter	Esa1	Pokholok *et al.* (20)
Methylation	H3R2me2a depleted in coding region		Kirmizis *et al.* (21)
Methylation	H3K4me3 enriched in promoter	Set1	Guillemette *et al.* (19) Pokholok *et al.* (20) Kirmizis *et al.* (21) Schulze *et al.* (22)
Methylation	H3K36me3 enriched in coding region		Pokholok *et al.* (20) Schulze *et al.* (22)
Methylation	H3K79me2 depleted in coding region		Schulze *et al.* (22)
Methylation	H3K79me3 depleted in coding region		Schulze *et al.* (22)
Ubiquitination	H2BK123ub enriched in coding region		Schulze *et al.* (22)
Histone variant	H2AZ depleted in promoter		Guillemette *et al.* (23)

## Utility and discussion

### The usage of YHMI

YHMI is a web tool for identifying the enriched/depleted histone modifications and the enriched histone/chromatin regulators in the input gene list. To use YHMI, users have to go through a three-step process (Figure 2). [Step 1] Users need to input a list of *N* genes, which will be analyzed by YHMI. Standard names, systematic names or aliases are all acceptable. [Step 2] Users need to define the sets of genes (in the yeast genome) whose promoters/coding regions contain specific histone modifications by setting the thresholds. For example, by setting ${\mathit{log}}_2\left(\mathrm{H}3\mathrm{K}9\mathrm{ac}/\mathrm{H}3\right)\ge 1$ (meaning 2-fold enrichment over the background) in the promoters, a set of 2129 yeast genes whose promoters contain H3K9ac could be defined. Then the expected ratio of promoters having H3K9ac in the yeast genome is equal to 0.32 (2129/6572). Further, by intersecting the input list of *N* genes and the set of 2129 genes, the number (denoted as *M*) of input genes whose promoters having H3K9ac can be calculated. Then the observed ratio of promoters having H3K9ac in the input list of genes is equal to *M*/*N*. Finally, the input list of *N* genes is said to be enriched with H3K9ac in the promoters if the observed ratio (*M*/*N*) is much larger than the expected ratio (2129/6572). The statistical significance is calculated using hypergeometric testing (30). [Step 3] Since YHMI tests the enrichment/depletion of many histone modifications (i.e. multiple hypotheses testing), users have to select a statistical method (Bonferroni correction or FDR) for multiple hypotheses correction and set the *P*-value threshold (0.01 is used as the default). The *P*-value threshold determines the statistical significance of the identified enriched/depleted histone modifications. The more stringent the *P*-value threshold, the higher the statistical significance of the identified enriched/depleted histone modifications. Therefore, if a user cannot find any enriched/depleted histone modifications for their gene list, they may want to loosen the *P*-value threshold to find some less statistically significant enriched/depleted histone modifications. Note that if a user wants to see all the results without defining the statistical significance level, he/she can choose ‘No *P*-value cutoff’.

**Figure 6:**
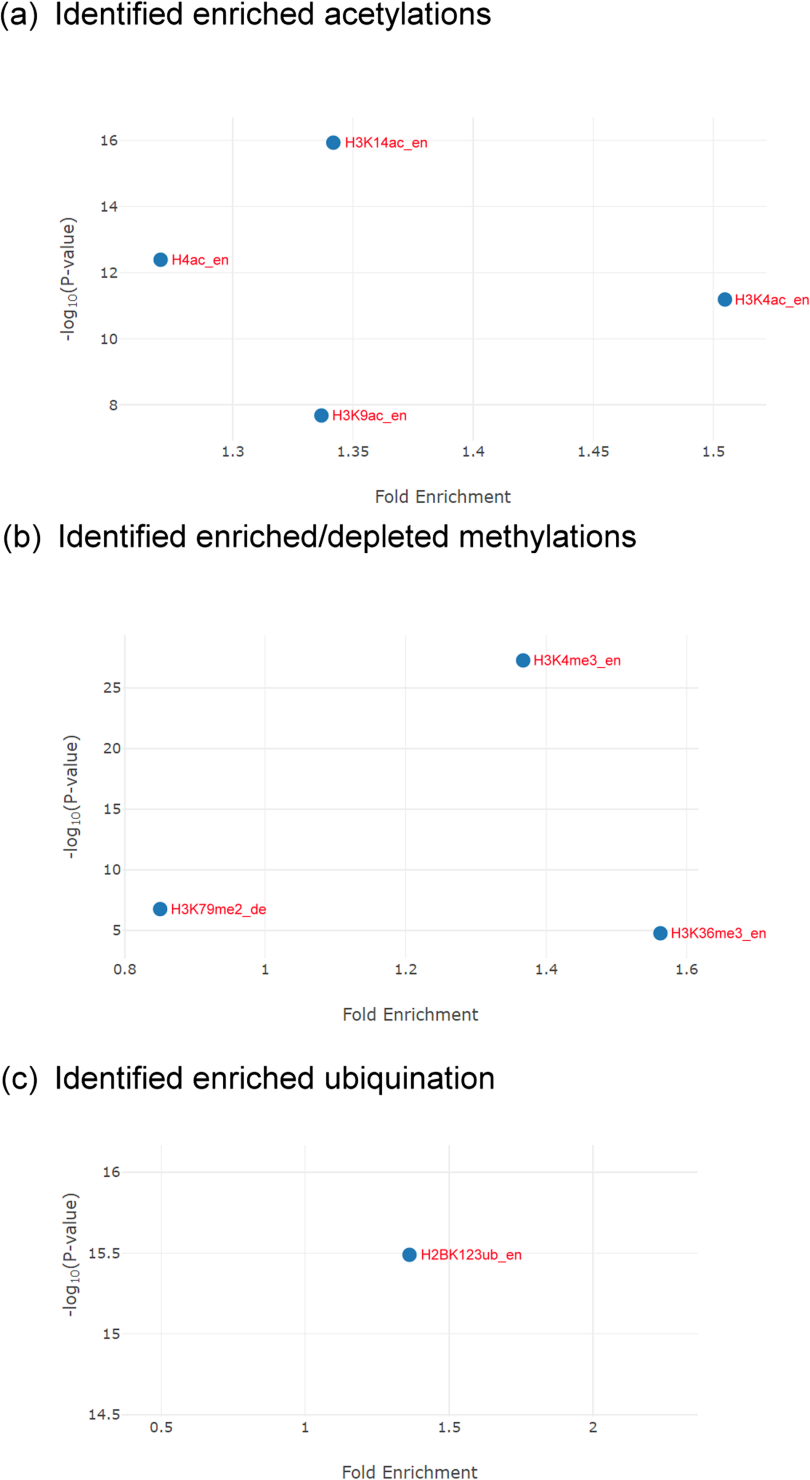
The identification results for the Esa1-targeting promoters. **(a)** YHMI identified four enriched acetylations (H3K4ac, H3K14ac, H3K9ac and H4ac). **(b)** YHMI identified two enriched methylations (H3K4me3 and H3K36me3) and one depleted methylation (H3K79me2). **(c)** YHMI identified one enriched ubiquitination (H2BK123ub).

After submission, YHMI will return the identification results that can be divided into two parts. First, the information of the user’s settings is shown. We allow users to download all the sets of genes containing specific histone modifications defined by the users for further investigation (Figure 3). Second, the identified enriched/depleted histone modifications (acetylation, methylation, phosphorylation, ubiquitination and histone variant) in the promoters/coding regions of the input gene list are shown as tables and figures. The table contains the following information: the name of the histone modification, trend (enriched/depleted), *P*-value, fold enrichment, observed ratio and expected ratio (Figure 4). Two kinds of figures (bar charts and volcano plots) are also provided for visualization (Figure 4). Two different bar charts (a fold enrichment bar chart and a *P*-value bar chart) are available. In the fold enrichment bar chart, the identified histone modifications are sorted by the fold enrichment values. In the *P*-value bar chart, the identified histone modifications are sorted by the *P*-values. The other kind of figure (the volcano plot) is a nice combination of the fold enrichment (x-axis) and *P*-value (y-axis). Moreover, the enriched histone/chromatin regulators in the promoters of the input gene list are provided as tables and figures (Figure 5).

### Two case studies

We use two case studies to show the high-quality and biological insight of YHMI’s identification results for the user’s input genes. Since several histone modifications are already known to be correlated with highly transcribed genes (19–23), we would like to check (in the first case study) whether YHMI can successfully identify these known histone modifications and their related enzymes. To do that, we retrieved a list of 174 highly transcribed (>50 mRNA/hr) genes under YPD condition from Holstege *et al.* (31). Strikingly, YHMI did successfully identify most histone modifications known to be enriched/depleted in highly transcribed genes (Table 3). For example, H3K4ac, H3K9ac and H3K14ac are identified to be enriched both in the promoters and the coding regions of the highly transcribed genes, consistent with existing knowledge (19). Moreover, YHMI successfully identifies the enrichment of Gcn5 [the known acetyltransferase which targets H3K4, H3K9 and H3K14 (19)] in the promoters of the highly transcribed genes, further supporting the biological significance of identifying the enrichment of H3K4ac, H3K9ac and H3K14ac. More examples could be found in Table 3. All these examples validate the high quality of YHMI’s identification results.

In the second case study, we used a list of 538 genes whose promoters are bound by Esa1 at 25°C (retrieved from Venters *et al.*’s study (18)) as the input to investigate the signature of histone modifications and histone/chromatin regulators of these Esa1-targeting promoters. Esa1 is a histone acetyltransferase specifically targeting histone H2A, H2AZ and H4 (17). Esa1 together with the other 12 subunits forms the NuA4 complex (17). YHMI identified several histone modifications and histone/chromatin regulators that are enriched in these Esa1-targeting promoters; in other words, the signature of histone modifications and regulators of these Esa1-targeting promoters.

**Table 4:** YHMI identified several histone/chromatin regulators that are related to NuA4 complex, SAGA complex or COMPASS complex

**Related to the complex**	**Identified regulator**	**Enrichment *P*-value**	**Fold enrichment** ^**a**^	**Observed ratio** ^**b**^	**Expected ratio** ^**c**^
NuA4	Esa1	0	12.22	100%	8.19%
NuA4	Rsc8	1.51E-21	1.59	51.3%	32.29%
NuA4	Eaf3	1.36E-16	2.16	21%	9.72%
NuA4	Hif1	1.09E-09	1.94	15.43%	7.97%
NuA4	Yaf9	2.31E-07	1.67	17.1%	10.24%
SAGA	Snf1	9.64E-51	2.56	42.57%	16.63%
SAGA	Rph1	2.22E-17	1.48	52.42%	35.36%
SAGA	Ahc1	4.15E-17	1.66	39.03%	23.57%
SAGA	Spt21	3.59E-12	2.28	13.75%	6.03%
SAGA	Snf2	7.03E-09	1.88	15.06%	8%
SAGA	Ada2	1.01E-08	1.63	22.3%	13.72%
SAGA	Snf5	2.13E-08	1.33	44.42%	33.44%
SAGA	Rxt1	3.57E-08	1.54	24.72%	16.02%
SAGA	Rvb2	2.02E-06	1.38	30.11%	21.88%
SAGA	Rpt6	1.96E-05	1.99	7.25%	3.64%
COMPASS	Set1	1.41E-38	6.7	11.52%	1.72%
COMPASS	Bre2	1.36E-06	1.34	35.13%	26.28%

a ^a^Fold enrichment = (Observed ratio) / (Expected ratio).

b ^b^Observed ratio = (number of input genes bound by the identified regulator) / (number of input genes).

c ^c^Expected ratio = (number of genes in the genome bound by the identified regulator) / (number of genes in the genome).

**Table 5:** YHMI’s identification results are robust against different data sources

**Histone Modification**	**Data Source**	**Trend**	***P*-value**	**Fold Enrichment**	**Observed Ratio**	**Expected Ratio**
H3K4me3	Guillemette 2011 (19)	Enriched	1.25E-41	1.93	95.40% (166/174)	49.33% (3242/6572)
H3K4me3	Kirmizis 2007 (21)	Enriched	4.18E-09	1.16	97.70% (170/174)	84.25% (5537/6572)
H3K4me3	Schulze 2011 (22)	Enriched	1.95E-08	1.16	97.13% (169/174)	84.04% (5523/6572)
H3K4me	Kirmizis 2007 (21)	Depleted	7.41E-39	0.19	10.92% (19/174)	57.59% (3785/6572)
H3K4me	Pokholok 2005 (20)	Depleted	7.19E-18	0.3	12.64% (22/174)	42.29% (2779/6572)
H3K36me3	Pokholok 2005 (20)	Enriched	6.23E-35	2.23	81.61% (142/174)	36.59% (2405/6572)
H3K36me3	Schulze 2011 (22)	Enriched	5.18E-14	1.36	90.80% (158/174)	66.81% (4391/6572)

For example, YHMI found significant enrichment of H3K4ac, H3K9ac, H3K14ac and H4ac in these Esa1-targeting promoters (Figure 6a). The enrichment of H4ac at these promoters is consistent with the function of Esa1 (17). Additionally, H3K4ac, H3K9ac and H3K14ac are also enriched in these promoters, indicating that the SAGA complex (which targets and acetylates H3K4, H3K9 and H3K14) might also act in these promoters along with Esa1 (32). Therefore, YHMI provides a testable hypothesis that SAGA might bind to promoters along with Esa1. This hypothesis awaits further experimental validation. Interestingly, YHMI found that H3K4me3 and H3K36me3 are also enriched in these Esa1-targeted promoters (Figure 6b). Therefore, YHMI provides another testable hypothesis that H3K4me3 and H3K36me3 might be important for regulating the acetyltransferase activity of NuA4. Indeed, several studies have shown that the methylated H3K4 and H3K36 interact with PHD and chromodomain within the NuA4 complex by using the GST pull-down, immunoprecipitation and protein array assays (9, 11, 12). Furthermore, the *set1Δ set2Δ* mutants or mutants with combined mutations of the PHD and chromodomains show defective acetyltransferases activity of NuA4 (33, 34). Additionally, an experiment has shown that the interaction of H3K4me3 with Tudor domain in Sgf29 within the SAGA complex is also essential for the acetyltransferase activity of SAGA (35). These experiments suggest the usefulness of YHMI.

YHMI also found that H2BK123ub is enriched in these Esa1-targeting promoters (Figure 6c). H2BK123ub is a transient histone mark, which is established by the Rad6/Bre1 ubiquitin ligase complex during transcription initiation and elongation (36). Previous studies have shown that H2BK123ub is essential for establishing H3K4me3 and H3K79me3 (37). In that sense, H3K4me3 and H3K79me3 are expected to be enriched in these Esa1-targeting promoters. YHMI successfully identified H3K4me3 enriched but did not see H3K79me3 enriched in these Esa1-targeting promoters (Figure 6b). Surprisingly, YHMI identified H3K79me2 depleted in these Esa1-targeting promoters (Figure 6b). The reason underlying H3K79me2 depletion is unclear. This awaits further experimental investigation. These examples illustrate how YHMI’s identification results can provide testable hypotheses for experimental investigation.

Consistent with H4ac, H3K4ac, H3K9ac and H3K14ac enrichment, YHMI found that several proteins within or interacting with the NuA4 complex (the H4 histone acetyltransferase complex) and the SAGA complex (the H3 histone acetyltransferase complex) are also enriched in these Esa1-targeting promoters (Table 4). These include Esa1, Eaf3, Rsc8, Yaf9 and Hif1 related to the NuA4 complex (38–40) and Ada2, Snf1, Ahc1, Rph1, Spt21, Snf2, Rxt1, SNF5, Rvb2 and Rpt6 related to the SAGA complex (41–46). Moreover, consistent with H3K4me3 enrichment, YHMI found Set1 and Bre2 in the COMPASS complex (the H3K4 methyltransferase complex) in these Esa1-targeting promoters (47). All these results show that YHMI is likely to return biologically meaningful results for the user’s input genes and provide user’s testable hypotheses for further investigation.

### Robustness of the identification results

Several histone modifications (e.g. H3K4me, H3K4me3 and H3K36me3) have ChIP-chip data in different sources and can be used to test whether YHMI’s identification results are robust against different data sources. For example, we tested whether the coding regions of the 174 highly transcribed genes (same input as in the case study 1) are enriched/depleted in H3K4me3. Strikingly, no matter which one of three ChIP-chip data sources of H3K4me3 is used, YHMI always returns the same trend (enrichment of H3K4me3). This observation is also true for H3K4me (depletion) and H3K36me3 (enrichment) shown in Table 5. These examples illustrate that the YHMI’s identification results are robust against different data sources.

### Comparison with our previously published YNA website

In 2014, we published YNA (26) which allows users to retrieve a list of yeast genes whose promoters and/or coding regions contain a user-specified combination of histone modifications (e.g. H3K4ac and H3K4me3). Since we published YNA, we received many requests for a reverse use (i.e. identifying the enriched histone modifications for a user’s gene list). This motivated us to develop YHMI. Compared to YNA, YHMI includes additional 17 histone modifications (9 histone acetylation, 6 histone methylation and 2 histone phosphorylation) from Weiner *et al.*’s ChIP-seq data (24). Moreover, users can download the lists of genes with a specific histone modification in YHMI. However, only YNA (but not YHMI) allows users to download a list of genes having a specific combination of histone modifications (e.g. H3K4ac and H3K4me3 in the promoters). To inform YHMI users about the related database YNA, we have provided a link and an introduction of YNA on the homepage of YHMI.

## Conclusion

In this study, we developed a web tool called YHMI. YHMI uses the ChIP-chip/ChIP-seq datasets of 32 histone modifications (15 histone acetylation, 13 histone methylation, 2 phosphorylation, 1 histone ubiquitination and 1 histone variant) and 83 histone/chromatin regulators. When a user inputs a gene list, YHMI will identify the enriched/depleted histone modifications in the promoters/coding regions and enriched histone/chromatin regulators in the promoters of the genes in the input list. The identification results are shown both in figures and tables. The high quality of YHMI’s results is validated by identifying most known histone modifications enriched/depleted in highly transcribed genes. The biological insight of YHMI’s results is demonstrated by generating experimentally testable hypotheses of novel histone modifications and their enzymes enriched in the target genes of the histone acetyltransferase Esa1. We believe that YHMI is a valuable tool for yeast biologists to do epigenetics research.

## Supplementary Material

Supplementary Data

## Notes

**Database URL:** http://cosbi4.ee.ncku.edu.tw/YHMI/
