# Supplementary material for: YHMI: a web tool to identify histone modifications and histone/chromatin regulators from a gene list in yeast
Source: Database (Oxford). 2018 Oct 29;2018:bay116. doi: 10.1093/database/bay116 (PMC6204766; doi:10.1093/database/bay116)
Supplement: Supplementary Data [file bay116_supp.zip › Supplementary Table 1_short.pdf]

Supplementary Table 1. The detailed information of the collected ChIP-chip/ChIP-seq datasets of 32 histone modifications

(15 histone acetylation, 13 histone methylation, 2 histone phosphorylation, 1 histone ubiquitination and 1 histone variant).

Due to the length constraint, we cannot show all the columns here.
See the complete Supplementary Table 1 at http://cosbi4.ee.ncku.edu.tw/YHMI/suptable2

Histone Acetylation	Data Value	Strain	Growth Condition	Reference Genome	Paper	Experimental Technology
Acetylation (H2AK5ac)	log_2(H2AK5ac/**Input**)	S288C (BY4741)	YPD	sacCer3 (R64)	Weiner et al., 2015	ChIP-seq
Acetylation (H3K4ac)	log_2(H3K4ac/H3)	S288C (BY4741)	YPD	sacCer3 (R64)	Guillemette et al., 2011	ChIP-chip
Acetylation (H3K9ac)	log_2(H3K9ac/H3)	W303a	YPD	sacCer3 (R64)	Pokholok et al., 2005	ChIP-chip
Acetylation (H3K14ac)	log_2(H3K14ac/H3)	W303a	YPD	sacCer3 (R64)	Pokholok et al., 2005	ChIP-chip
Acetylation (H3K14ac)	log_2(H3K14ac/H3)	W303a	YPD adding H_2O_2	sacCer3 (R64)	Pokholok et al., 2005	ChIP-chip
Acetylation (H3K18ac)	log_2(H3K18ac/Input)	S288C (BY4741)	YPD	sacCer3 (R64)	Weiner et al., 2015	ChIP-seq
Acetylation (H3K23ac)	log_2(H3K23ac/Input)	S288C (BY4741)	YPD	sacCer3 (R64)	Weiner et al., 2015	ChIP-seq
Acetylation (H3K27ac)	log_2(H3K27ac/Input)	S288C (BY4741)	YPD	sacCer3 (R64)	Weiner et al., 2015	ChIP-seq
Acetylation (H3K56ac)	log_2(H3K56ac/Input)	S288C (BY4741)	YPD	sacCer3 (R64)	Weiner et al., 2015	ChIP-seq
Acetylation (H4ac)	log_2(H4ac/H3)	W303a	YPD	sacCer3 (R64)	Pokholok et al., 2005	ChIP-chip
Acetylation (H4ac)	log_2(H4ac/H3)	W303a	YPD adding H_2O_2	sacCer3 (R64)	Pokholok et al., 2005	ChIP-chip

Acetylation (H4K5ac)	$\log_2$(H4K5ac/Input)	S288C (BY4741)	YPD	sacCer3 (R64)	Weiner et al., 2015	ChIP-seq
Acetylation (H4K8ac)	$\log_2$(H4K8ac/Input)	S288C (BY4741)	YPD	sacCer3 (R64)	Weiner et al., 2015	ChIP-seq
Acetylation (H4K12ac)	$\log_2$(H4K12ac/Input)	S288C (BY4741)	YPD	sacCer3 (R64)	Weiner et al., 2015	ChIP-seq
Acetylation (H4K16ac)	$\log_2$(H4K16ac/Input)	S288C (BY4741)	YPD	sacCer3 (R64)	Weiner et al., 2015	ChIP-seq

**Histone Phosphorylation, Ubiquitination and Variant**	**Data Value**	**Strain**	**Growth Condition**	**Reference Genome**	**Paper**	**Experimental Technology**
Phosphorylation (H2AS129ph)	$\log_2$(H2AS129ph/Input)	S288C (BY4741)	YPD	sacCer3 (R64)	Weiner et al., 2015	ChIP-seq
Phosphorylation (H3S10ph)	$\log_2$(H3S10ph/Input)	S288C (BY4741)	YPD	sacCer3 (R64)	Weiner et al., 2015	ChIP-seq
Ubiquitination (H2BK123ub)	MAT score (H2BK123ub/Input)	Other (SLJ001, YSN545, YSN763, YSN4, YSN18)	YPD	sacCer3 (R64)	Schulze et al., 2011	ChIP-chip
Histone Variant (H2AZ)	$\log_2$(H2AZ/H2B)	Other (BGY02)	YPD	sacCer3 (R64)	Guillemette et al., 2005	ChIP-chip

**Histone Methylation**	**Data Value**	**Strain**	**Growth Condition**	**Reference Genome**	**Paper**	**Experimental Technology**
Methylation (H3R2me2a)	$\log_2$(H3R2me2a/H3)	S288C	YPD	sacCer3 (R64)	Kirmizis et al., 2007	ChIP-chip

Methylation (H3K4me)	log_2(H3K4me/H3)	W303a	YPD	sacCer3 (R64)	Pokholok et al., 2005	ChIP-chip
Methylation (H3K4me2)	log_2(H3K4me2/H3)	W303a	YPD	sacCer3 (R64)	Pokholok et al., 2005	ChIP-chip
Methylation (H3K4me3)	log_2(H3K4me3/H3)	S288C	YPD	sacCer3 (R64)	Guillemette et al., 2011	ChIP-chip
Methylation (H3K36me)	log_2(H3K36me/Input)	S288C (BY4741)	YPD	sacCer3 (R64)	Weiner et al., 2015	ChIP-seq
Methylation (H3K36me2)	log_2(H3K36me2/Input)	S288C (BY4741)	YPD	sacCer3 (R64)	Weiner et al., 2015	ChIP-seq
Methylation (H3K36me3)	log_2(H3K36me3/H3)	W303a	YPD	sacCer3 (R64)	Pokholok et al., 2005	ChIP-chip
Methylation (H3K79me)	log_2(H3K79me/Input)	S288C (BY4741)	YPD	sacCer3 (R64)	Weiner et al., 2015	ChIP-seq
Methylation (H3K79me2)	**MAT score** (H3K79me2/Input)	W303	YPD	sacCer3 (R64)	Schulze et al., 2011	ChIP-chip
Methylation (H3K79me3)	MAT score (H3K79me3/Input)	W303	YPD	sacCer3 (R64)	Schulze et al., 2011	ChIP-chip
Methylation (H4R3me)	log_2(H4R3me/Input)	S288C (BY4741)	YPD	sacCer3 (R64)	Weiner et al., 2015	ChIP-seq
Methylation (H4R3me2s)	log_2(H4R3me2s/Input)	S288C (BY4741)	YPD	sacCer3 (R64)	Weiner et al., 2015	ChIP-seq
Methylation (H4K20me)	log_2(H4K20me/Input)	S288C (BY4741)	YPD	sacCer3 (R64)	Weiner et al., 2015	ChIP-seq

"Input" means the control experiment, which is the ChIP-chip/ChIP-seq experiment without using any anti-histone modification (e.g. anti-H3K79me2) antibody.

MAT stands for Model-based Analysis of Tiling-arrays, which is an algorithm for reliably detecting enriched regions. The higher the MAT score, the higher the enrichment.

References

1. Pokholok,D.K., Harbison,C.T., Levine,S. *et al.* (2005) Genome-wide map of nucleosome acetylation and methylation in yeast. *Cell* , **122**, 517-527.
2. Guillemette,B., Bataille,A.R., Gevry,N. *et al.* (2005) Variant histone H2A.Z is globally localized to the promoters of inactive yeast genes and regulates nucleosome positioning. *PLoS Biol.* , **3**, e384.
3. Kirmizis,A., Santos-Rosa,H., Penkett,C.J. *et al.* (2007) Arginine methylation at histone H3R2 controls deposition of H3K4 trimethylation. *Nature* , **449**, 928-932.
4. Schulze,J.M., Hentrich,T., Nakanishi,S. *et al.* (2011) Splitting the task: Ubp8 and Ubp10 deubiquitinate different cellular pools of H2BK123. *Genes Dev.* , **25**, 2242-2247.
5. Guillemette,B., Drogaris,P., Lin,H.H. *et al.* (2011) H3 lysine 4 is acetylated at active gene promoters and is regulated by H3 lysine 4 methylation. *PLoS Genet.* , **7**, e1001354.
6. Weiner,A., Hsieh, TH, Appleboim,A. et al. (2015) High-Resolution Chromatin Dynamics during a Yeast Stress Response. Mol. Cell, 58(2), 271-386.
