# Supplementary material for: YHMI: a web tool to identify histone modifications and histone/chromatin regulators from a gene list in yeast
Source: Database (Oxford). 2018 Oct 29;2018:bay116. doi: 10.1093/database/bay116 (PMC6204766; doi:10.1093/database/bay116)
Supplement: Supplementary Data [file bay116_supp.zip › Supplementary Table 2.pdf]

Supplementary Table 2.

This table provides the number of genes whose promoters are bound by 83 histone/chromatin regulators under normal (25 oC) or acute heat-shock (37 oC) conditions.

See the systematic names of the target genes at http://cosbi4.ee.ncku.edu.tw/YHMI/supplementary

histone/chromatin regulators	# of target genes whose promoters are bound by this regulator
Ada2_25C	360
Ada2_37C	902
Ahc1_25C	1549
Ahc1_37C	1117
Aor1_25C	2102
Aor1_37C	2352
Arp6_25C	1506
Arp6_37C	1445
Asf1_25C	1203
Asf1_37C	989
Bre1_25C	1306
Bre1_37C	1530
Bre2_25C	1624
Bre2_37C	1727
Brn1_25C	993
Brn1_37C	1411
Chd1_25C	329
Chd1_37C	187
Chz1_25C	0
Chz1_37C	802
Cyc8_25C	2892
Cyc8_37C	2764
Dot1_25C	1652
Dot1_37C	1099
Eaf3_25C	639
Eaf3_37C	749
Epl1_25C	1234
Epl1_37C	842
Esa1_25C	538
Esa1_37C	2974
Gcn5_25C	2333
Gcn5_37C	2638
Hda1_25C	1619
Hda1_37C	2572
Hho1_25C	631
Hho1_37C	523
Hht1_25C	1638
Hht1_37C	1286
Hht2_25C	760
Hht2_37C	132

Hif1_25C	524
Hif1_37C	748
Hos1_25C	1649
Hos1_37C	2120
Hos2_25C	889
Hos2_37C	992
Hos3_25C	649
Hos3_37C	731
Hpa3_25C	1160
Hpa3_37C	1391
Hta2_25C	0
Hta2_37C	0
Htb1_25C	1259
Htb1_37C	990
Htb2_25C	2090
Htb2_37C	2264
Htz1_25C	4665
Htz1_37C	4727
Ino80_25C	3546
Ino80_37C	3749
Ioc2_25C	2420
Ioc2_37C	2335
Ioc3_25C	1223
Ioc3_37C	2431
Ioc4_25C	688
Ioc4_37C	799
Irc20_25C	1750
Irc20_37C	2427
Irr1_25C	706
Irr1_37C	647
Isw1_25C	2677
Isw1_37C	3159
Isw2_25C	2196
Isw2_37C	2138
Itc1_25C	3524
Itc1_37C	3161
Jhd1_25C	398
Jhd1_37C	878
Jhd2_25C	598
Jhd2_37C	978
Nap1_25C	0
Nap1_37C	7
Nhp6a_25C	1514
Nhp6a_37C	2578
Otu1_25C	1097
Otu1_37C	1234
Rad6_25C	1411
Rad6_37C	1527
Rpd3_25C	2864
Rpd3_37C	2244
Rph1_25C	2324
Rph1_37C	2310

Rpt1_25C	1671
Rpt1_37C	1254
Rpt6_25C	239
Rpt6_37C	106
Rsc1_25C	86
Rsc1_37C	2265
Rsc2_25C	1050
Rsc2_37C	2000
Rsc4_25C	222
Rsc4_37C	7
Rsc8_25C	2122
Rsc8_37C	2110
Rsc9_25C	3537
Rsc9_37C	4601
Rtt109_25C	409
Rtt109_37C	445
Rvb1_25C	1239
Rvb1_37C	1371
Rvb2_25C	1438
Rvb2_37C	1418
Rxt1_25C	1053
Rxt1_37C	1635
Rxt2_25C	3011
Rxt2_37C	2886
Sas4_25C	1055
Sas4_37C	1223
Set1_25C	113
Set1_37C	38
Set2_25C	1771
Set2_37C	1834
Sif2_25C	820
Sif2_37C	692
Sin3_25C	302
Sin3_37C	337
Sir2_25C	2006
Sir2_37C	1369
Sir3_25C	461
Sir3_37C	642
Snf1_25C	1093
Snf1_37C	983
Snf2_25C	526
Snf2_37C	557
Snf4_25C	1170
Snf4_37C	1322
Snf5_25C	2198
Snf5_37C	1579
Snf7_25C	374
Snf7_37C	746
Spt21_25C	396
Spt21_37C	0
Spt2_25C	1837
Spt2_37C	1807

Swc1_25C	2829
Swc1_37C	2496
Swi3_25C	2493
Swi3_37C	2408
Swi5_25C	987
Swi5_37C	1125
Swr1_25C	4180
Swr1_37C	4920
Tup1_25C	257
Tup1_37C	663
Ubp8_25C	169
Ubp8_37C	192
Vps72_25C	4086
Vps72_37C	4918
Yaf9_25C	713
Yaf9_37C	673
Ycg1_25C	872
Ycg1_37C	1197
Ycs4_25C	2012
Ycs4_37C	2342
Yng1_25C	1000
Yng1_37C	1418
